# Prompt-Driven Multimodal Segmentation with Dynamic Fusion for Adaptive and Robust Medical Imaging with Applications to Cancer Diagnosis

**DOI:** 10.3390/cancers17223691

**Published:** 2025-11-18

**Authors:** Shatha Abed Alsaedi, Hossam Magdy Balaha, Mohamed Farsi, Majed Alwateer, Moustafa M. Aboelnaga, Mohamed Shehata, Mahmoud Badawy, Mostafa A. Elhosseini

**Affiliations:** 1 Department of Computer Science, College of Computer Science and Engineering, Taibah University, Yanbu 46421, Saudi Arabia; saasaedi@taibahu.edu.sa (S.A.A.);; 2 Bioengineering Department, J.B. Speed School of Engineering, University of Louisville, Louisville, KY 40292, USA; 3 Computers and Control Systems Engineering Department, Faculty of Engineering, Mansoura University, Mansoura 35516, Egypt; 4 Department of Information Systems, College of Computer Science and Engineering, Taibah University, Yanbu 46421, Saudi Arabia; 5 Department of Software Engineering, SolarWinds Company, Holandská 873, 639 00 Brno, Czech Republic; 6 Computer Science Program, Department of Business, Midway University, Midway, KY 40347, USA; 7 Department of Computer Science and Information, Applied College, Taibah University, Medinah 41461, Saudi Arabia

**Keywords:** multimodal segmentation, prompt-driven AI, cross-modal fusion, medical image analysis

## Abstract

Background/Objectives: Medical image segmentation is a crucial task for diagnosis, treatment planning, and monitoring of cancer; however, it remains one of the toughest nuts to crack for Artificial Intelligence (AI)-based clinical applications. Deep-learning models have shown near-perfect results for narrow tasks such as single-organ Computed Tomography (CT) segmentation. Still, they fail to deliver under practicality, in which cross-modality robustness and multi-organ delineation are essential (e.g., liver Dice dropping to 0.88 ± 0.15 in combined CT-MR scenarios). That fragility exposes two structural gaps: (i) rigid task-specific architectures, which are not flexible enough to adapt to various clinical instructions, and (ii) the assumption that a universal loss function is best in all cancer imaging applications. Methods: A novel multimodal segmentation framework is proposed that combines natural language prompts and high-fidelity imaging features through Feature-wise Linear Modulation (FiLM) and Conditional Batch Normalization, enabling a single model to adapt dynamically across modalities, organs, and pathologies. Unlike preceding systems, the proposed approach is prompt-driven, context-aware, and end-to-end trainable to ensure alignment between computational adaptability and clinical decision-making. Results: Extensive evaluation on the Brain Tumor Dataset (cancer-relevant neuroimaging) and the CHAOS multi-organ challenge demonstrates two key insights: (1) while Dice loss remains optimal for single-organ tasks, (2) Jaccard (IoU) loss outperforms when multi-organ, cross-modality divides cancer segmentation boundaries. Empirical evidence has thus been offered that optimality of a loss function is task- and context-dependent and not universal. Conclusions: The design framework’s principles directly address what is documented in workflow requirements and display capabilities that may connect algorithmic innovation with clinical utility once validated through prospective clinical trials.

## Simple Summary

Accurate segmentation of tumors and organs from medical images is vital for cancer diagnosis and treatment, yet current AI tools often fail when applied across different imaging types (e.g., CT and MRI) or when multiple organs must be segmented simultaneously. This study introduces a flexible AI system that listens to natural language instructions (such as “segment the meningioma”) and adapts its behavior accordingly, without retraining. By dynamically fusing textual and imaging data, the model achieves high accuracy in both single- and multi-organ tasks. Importantly, the study shows that the best mathematical objective (loss function) depends on the task: Dice works best for single tumors, while Jaccard (IoU) is superior for complex, multi-organ cases. These insights help bridge the gap between AI research and real-world clinical use.

## 1. Introduction

The value of automated medical image segmentation is apparent in modern healthcare for vital diagnosis, meticulous monitoring, and efficacious treatment planning [1,2]. The yearly load of advanced medical imaging modalities (hundreds of millions of diagnostic scans processed in the USA alone) has grown tremendously as it has made manual interpretations long, arduous, and highly variable due to inter- or intra-expert inconsistencies [3,4]. This, therefore, has created bottlenecks, which would be critical in delaying necessary diagnoses and overall effective clinical workflows due to human effort [5].

The promising potential of deep learning (DL) techniques for automated (and high-fidelity) segmentation generally relies on fully convolutional architectures; however, widespread clinical utility of these methods is still hampered by several persisting fundamental limitations [6]. To begin with, the development of extensively annotated medical datasets remains a painstaking and resource-hungry task, which often leads to overfitting of the model and, hence, hampers robust generalization across multiple patient populations [2,7]. Also, extreme class imbalance situates target lesions (or organs) to cover a tiny proportion of the image, for example, healthy tissue occupying an overwhelming ≈99.12% in brain tumor segmentation, which biases algorithms and leads to poor detection of truly relevant anomalies [5,8].

Yet another barrier that restricts current deep learning approaches from being applicable in genuine medicine is the issues associated with robustness and adaptation [1,9]. Although these architectures exhibit highly accurate performance in unimodal testing (e.g., 0.98±0.00 Dice coefficients for CT liver segmentation), their efficacy drops steeply in the presence of challenges in cross-modality data handling (e.g., 0.88±0.15 in Dice coefficient concerning liver segmentation) and in processes of multi-organ segmentation (generating an approximated 5% lower income in revenue than organ-specific ones). This profoundly decreases the maximum confidence level in a diagnosis because it does not adhere to the required configurability across several clinical scenarios, integrating variations in image quality thrown back from acquisition protocols and patient anatomies [10].

Overcoming the prohibitively large computational burden of any viable 3D convolutional architecture has become rather essential to implement real-world bulk applications of any 3D-based CAD solution. These particular patients who are desperate for anatomical data for drastic life-altering conditions could be immensely affected by the technical and practical aspects [5,7]. Delays and uncertainties subject to segmentation procedures could be fatal through cancer staging and liver transplant assessment, through specific cardiac and neurologic pathology evaluations, to name a few, and possibly urgent treatment-requiring cases, such as diabetic retinopathy or soft tissue sarcomas [10]. In a way, this lack of flexible, intuitive image analysis tools indirectly foreclosed the opportunity to plan patients’ therapies on time, further negating millions of clinical outcomes each year across the world. For some reason, this has created an urgent need for state-of-the-art precision diagnostic tools for approximately 6.7 million individuals aged 65 and older in the US with Alzheimer’s disease [1,9].

Overcoming some of these daunting challenges is necessary. We require robust yet customizable deep learning (DL) solutions that can generalize innovation to handle a variety of challenges, such as learning from less data, class imbalance, and diverse problems in imaging [11,12]. Proper fine-tuning of loss functions that emphasize medical imaging tasks (for example, appropriately weighing false positives against false negatives) has consistently improved segmentation accuracy, aiding such methods in boosting performance in challenging medical imaging tasks by up to about 12% [7,10]. The fusion of DL and other modalities, such as PET-CT-MRI, appears to hybridize smoothly and operate quite efficiently with an average Dice coefficient of 85% in some of the more challenging applications, such as soft tissue sarcoma segmentation (a great deal improved over single-modality applications). Now the platform is set to work toward establishing strong, efficient, and fair DL solutions that could overturn present challenges, reconnecting the chasm between the research environment and a real-world medical application [5,10].

Collaborative modeling should not only be the aggregation of larger, more generalizable datasets but also model development itself, and the architecture should support robust patient prediction based on advanced imaging analysis techniques [3,13,14]. Such development looks ahead to the normalization of clinical decisions in a modernized and eco-sensitive manner towards better clinical outcome orientation and ushering in an era of trustworthiness and efficiency for all concerning approaches to medical image analysis for the joint empowerment of precision medicine delivery for all at-risk population groups [15,16,17].

This study presents a versatile teachable multimodal segmentation architecture model combining high-fidelity multi-scale visual features of medical data and semantically rich natural language instructions to obtain unprecedented levels of clinical adaptability and precision. Its main novelty lies in the dynamic context-aware fusion of textual prompts with visual features through an advanced Feature-wise Linear Modulation (FiLM) layer with Conditional Batch Normalization and dual attention mechanisms (SE and CBAM), which allow a single model to perform multiple segmentation tasks entirely based on user-defined commands, without modifying its architecture and without task-specific retraining.

We validate this architecture on two different clinically relevant benchmarks, the Brain Tumor Dataset and the CHAOS challenge dataset, demonstrating state-of-the-art performance and providing one important context-sensitive guideline: the Dice loss is optimal for single-organ, single-modality tasks such as brain tumor delineation, whereas the Jaccard (IoU) loss becomes the best and most robust choice for complex multi-organ segmentation under the extreme challenges of cross-modality generalization. Supported with rigorous quantitative results and qualitative inference, the above finding constitutes an essential result concerning the selection of loss functions for medical image analysis. The work has three core contributions:

- Adaptive framework: We design an end-to-end prompt-driven multimodal segmentation system, fusing natural language and medical imaging features through FiLM and Conditional Batch Normalization for context-aware adaptability.
- Loss-function context-dependent insight: We systematically evaluate Dice versus Jaccard losses across single-organ and multi-organ, cross-modality tasks, empirically proving that loss selection is task-dependent rather than universal.
- Clinical translation: We provide a robust, generalizable solution that bridges algorithmic innovation and clinical need, delivering accurate and interpretable segmentations—an essential step toward scalable deployment in real diagnostic workflows.

It is worth noting that this study is purely computational and does not involve wet-lab experiments, patient recruitment, or clinical outcome analysis. All experiments were conducted in silico using publicly available, de-identified medical imaging datasets (BraTS, CHAOS). No biological reagents, assays, or survival modeling (e.g., Kaplan–Meier, ROC) are applicable to this work.

The paper is organized as follows: Section 2 reviews related work. In Section 3, the proposed end-to-end trainable multimodal segmentation architecture is described. In Section 4, the experiments are presented, and the results are analyzed. In Section 5, the limitations are detailed. Section 6 concludes the paper, and future work is introduced.

## 2. Related Studies

Deep learning has advanced medical image segmentation that helps aid computer-assisted diagnosis, therapy, and surgical planning with remarkable performance. Yet, a consensus on optimal techniques and approaches remains elusive. For instance, Conze et al. reviewed the current trends, highlighted the different developments (since the U-Net-inspired networks) comprehensively, and noted the DL applicability across multiple medical imaging modalities and clinical contexts [18]. Additionally, Liu et al. listed the recent progress, merits, and drawbacks while underscoring persistent challenges (such as limited dataset sizes, low image resolution, and insufficient segmentation accuracy) for clinical requirements [10]. Similarly, Hesamian et al. appraised the popular DL techniques and examined many network architectures (i.e., 2D, 2.5D, 3D CNNs, U-Nets, V-Nets) and training techniques, while identifying key challenges (including limited annotated data, class imbalance, overfitting, and gradient vanishing) [19]. Also, thematic surveys by Wang et al. detailed the supervised (and weakly supervised) learning approaches where they elaborated different backbone networks, loss functions, data augmentation, and emerging directions (including neural architecture search and medical transformers) [8].

Specifically addressing architectural efficacy and loss function optimization, Furtado investigated popular loss functions and their variations for DL-based multiclass medical image segmentation [2]. The study found that DeepLabV3 consistently outperformed U-Net and FCN with Dice loss, generally exhibiting superior performance and improving scores by 1 to 6 percentage points (pp) over cross entropy. Also, customized false positive/false negative weighting and zero-weighting of the background class significantly enhanced segmentation and boosted scores by up to 12 pp and 6 pp, respectively. Furthermore, in [20], the authors created a 3D fully convolutional network (FCN) (notably, a custom 3D U-Net-like architecture) for automated semantic multi-organ segmentation in CT images, achieving an average Dice score of 89.3±6.5% with fast inference times. Lai [21] investigated CNN architectures for patch-based 3D hippocampal segmentation and concluded that a tri-planar approach offered the most favorable balance between accuracy (test error ≈8.6%) and training efficiency, despite a full 3D architecture achieving a slightly better patch classification error of ≈7.4%.

Beyond single-modality advancements, using multimodal data presents a significant research thrust. For example, Guo et al. [9] investigated multimodal fusion schemes for supervised image segmentation. They proposed a deep CNN system for soft tissue sarcoma lesions. Their patch-based approach showed that multimodal fusion networks consistently surpassed single-modal networks in performance (e.g., PET/CT/T2 achieving 85% average DICE vs. single-modality T2 at 80%), indicating robustness to image degradation. Moreover, Zhou et al. elaborated on the different DL methodologies available for multimodal medical image segmentation by examining various fusion approaches (input-level, layer-level, and decision-level) and emphasizing the mandatory role of multimodal information [7]. Unlike those fusion modalities, the CHAOS challenge by Kavur et al. indicated the incessant problems concerning the cross-modality and multi-organ generalization [22]. While the volumetric performances of single-modality liver segmentation networks were particularly high (e.g., CT DICE: 0.98 ± 0.00; MR DICE: 0.95 ± 0.01), the performance witnessed sharp declines in cross-modality settings, especially in the case of the merged multi-organ segmentation task (liver cross-modality DICE: 0.88 ± 0.15), further underscoring the need for solid solutions in real-world clinical evidence applications.

Interactive segmentation techniques have continued to elevate their robustness and attempted real clinical integration. Wang et al. constructed BIFSeg, an interactive DL framework incorporating CNNs into a bounding box and scribble-based pipeline [8]. The framework displayed much greater robustness and gave superior segmentation accuracy (for example, with supervised fine-tuning, the Dice score improved by 1.3–5 pp), decreased user interaction, and took much less time than its ancestors. Another set of tools, MIScnn, introduced by Muller and Kramer, also tries to ease the development of medical image segmentation pipelines [6]. This open-source Python v3 library contains an intuitive API *that integrates* data I/O, preprocessing, augmentation, and a library of newly developed models *such as* 3D U-Net. With great success, it has shown that establishing high-performing predictors in record time is feasible (for example, median Dice coefficients ≈0.9544 for kidneys and ≈0.7912 for tumors).

The most recent rise of foundation models has greatly influenced medical image segmentation. Models like Segment Anything Model (SAM) [23] and its medical adaptation, MedSAM [24], have shown amazing zero-shot and few-shot performance by massively pretraining on natural images and fine-tuning on medical ones. These models provide strong general-purpose segmentation tools that can be queried using either points or boxes. Unfortunately, they often behave as black boxes and may not permit a natural approach for dynamically and context-aware fusion of multiple modalities or for optimizing an explicit task-specific loss function. This paper extends that work to present a framework whereby natural language prompts directly condition the model’s internal feature representation, utilizing FiLM and CBN, to create a more interpretable and completely end-to-end trainable system aimed at the subtleties of multi-organ, cross-modality cancer imaging.

### Research Gaps

Medical image segmentation has reached a new height with the introduction of deep learning. However, today, these systems remain extremely brittle when used in clinical workflows. State-of-the-art models score near perfection in controlled environments (e.g., segmentation of a single organ on CT images (Dice > 0.98)); however, in practical situations where cross-modality robustness is required, the same models fail (e.g., Dice scores dropping to 0.88 ± 0.15 for CT-MR integration) and for multi-organ generalization, as demonstrated by findings in the CHAOS challenge. This phenomenon of brittleness is due to two bottlenecks that have remained inadequately addressed:

- Rigid task-specificity: Models are locked to static datasets and narrow problem definitions, lacking the dynamic adaptability to follow diverse, natural language clinical instructions.
- Universal loss function: While the standard assumption is to have only one loss (typically the Dice loss) that treats all tasks in the same way, our results demonstrate that the performance of losses varies greatly between tasks, anatomical areas, and modalities. Dice loss is best for delineating single organs, while multi-organ delineations still have a very weak performance, and the Jaccard (IoU) shows far better stability.

These gaps point to a fundamental challenge: the absence of a unified, multimodal, prompt-driven framework that can flexibly integrate linguistic and imaging cues while dynamically optimizing its loss strategy, thereby aligning computational design with the agility of clinical decision-making. Table 1 depicts the Gap-Contribution Mapping.

## 3. Methods

**Figure 1:**
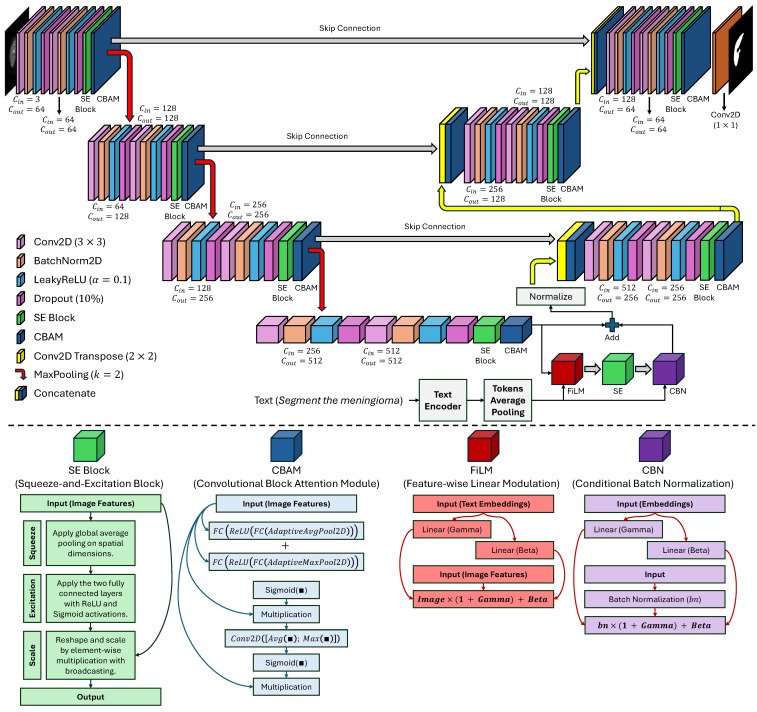
Schematic diagram of the designed U-Net encoder and decoder.

This study proposes an end-to-end trainable multi-modal segmentation framework (see Figure 1) meant to address the interlinked and perennial challenges of extreme class imbalance, limited data availability, and extreme model nonadaptability to a variety of context-specific clinical queries in automated medical image analysis. This framework moves far away from the standard unimodal DL approach by incorporating multi-scale high-fidelity visual representations derived from multi-modal data synergistically with semantically rich natural language instructions that encode subtle intents from a clinical user. The architecture is conceived as a tightly-coupled hierarchy of four interdependent, functionally-specialized modules or components: (1) a hierarchical, attention-enhanced image encoder extracting and refining spatial and contextual features, in an incremental manner, from raw pixel data, (2) a pre-trained and fine-tuned text encoder performing the transformation of user-given, domain-specific linguistic commands into dense context-sensitive semantic embeddings, (3) an advanced conditional cross-modal fusion module which modulates the visual feature space in a context-dependent manner, and (4) a skip-connected attention-based guided decoder responsible for reconstructing a high-resolution, anatomically precise segmentation mask.

The designs are dominated by a cognitive clinical model whereby a radiologist not only detects a lesion but actively questions and answers diagnostic questions. The proposed model achieves this human-like adaptability by the segmentation being conditioned on natural language, thus allowing a single architecture to segment glioma, meningioma, or pituitary tumors, as per the prompt, without any model retraining or architecture modification specific to the task. The entire end-to-end system is trained under a composite clinically motivated loss with explicit penalties for volumetric error, boundary discontinuity, and topologic error, thus resulting in segmentations that are not only quantitatively accurate but also qualitatively consistent with the underlying biological reality, which makes them very clinically relevant and helpful in decision-making.

### 3.1. Image Encoder: Hierarchical Multi-Scale Feature Extraction with Attention

The image encoder is the minimal part of the eye that constructs from the raw input, a high-dimensional $I\in R^{H\times W\times C}$, feature representation that is rich in hierarchical and semantic meaning, where *H* and *W* refer to the spatial dimensions of the image, while *C* is the number of input channels that can refer to different data sequences (e.g., T1-weighted, T2-weighted, FLAIR, or the combinations of these), where each channel provides complementary information about tissue contrast and pathology. The encoder is arranged as a modified U-Net backbone, a thorough design that is aimed at saving the detailed structural entities needed for the depiction of the subtle and irregular tumor margins, at the same time, it is absorbing the high-level semantic abstractions of the overall tissue context. The encoding process is carried out in four distinctive stages, each formed by a residual double convolutional block, followed by the cascading of spatial and channel-wise attention mechanisms, and finally, downsampling to reduce spatial resolution while increasing feature depth. Let $E_{l}$ denote the feature map at the *l*-th encoding stage, where l∈{1,2,3,4}, and let $E_{0}=I$ serve as the input picture. The core convolutional operation at each stage can be defined in Equation (1), where ${\mathrm{Downsample}}_{l-1}$ constitutes a max-pooling procedure utilizing a kernel size of 2×2 and a stride of two, thus diminishing the spatial dimensions by half and effectively increasing the receptive field of subsequent layers.

(1) $$E_{l}={\mathrm{ConvBlock}}_{l}\left({\mathrm{Downsample}}_{l-1}\left(E_{l-1}\right)\right)$$

The ${\mathrm{ConvBlock}}_{l}$ is a residual unit comprising two consecutive 3×3 convolutional layers, where each is subsequently accompanied by batch normalization (BN) to enhance training stability and a LeakyReLU activation function with a negative slope of 0.1 to alleviate the “dying ReLU” issue and improve the gradient flow. Crucially, to enhance feature discriminability and suppress irrelevant activations arising from noise or non-pathological structures, each ${\mathrm{ConvBlock}}_{l}$ is augmented with two distinct, complementary attention mechanisms. First, a Squeeze-and-Excitation (SE) block [25] recalibrates channel-wise feature responses by capturing global dependencies across the entire feature map. Given a feature map $F\in R^{C\times H^{\prime }\times W^{\prime }}$, the SE operation is computed using Equation (2), where GAP(·) denotes global average pooling, which consolidates spatial information into a singular description for each channel.

(2) $$z=\mathrm{GAP}\left(F\right)=\frac{1}{H^{\prime }\times W^{\prime }}\times \sum_{i=1}^{H^{\prime }}\sum_{j=1}^{W^{\prime }}F_{:,i,j}\in R^{C}$$

This descriptor is then passed through two fully connected layers with a bottleneck reduction ratio r=16 to model non-linear channel interactions, as shown in Equation (3), where $W_{1}\in R^{C/r\times C}$ and $W_{2}\in R^{C\times C/r}$ are learnable weight matrices, and σ is the Sigmoid function.

(3) $$u=\sigma \left(W_{2}\;\cdot \;\mathrm{ReLU}\left(W_{1}\;\cdot \;z\right)\right)\in R^{C}$$

The resulting attention weights u are then broadcast. Element-wise multiplied with the original feature map to produce a recalibrated output using Equation (4), where ⊙ denotes element-wise multiplication.

(4) $$F_{\mathrm{SE}}=u⊙F$$

This allows the model to amplify the response of channels most relevant to the presence of pathology and suppress those associated with background or noise. Second, a Convolutional Block Attention Module (CBAM) [26] is applied to refine the spatial and channel-wise features, providing a more localized form of attention. The CBAM computes a spatial attention map $M_{s}$ by concatenating the average and maximum pooled feature maps along the channel dimension and passing them through a single 7×7 convolutional filter to capture spatial dependencies using Equation (5), where $F_{7\times 7}$ is the convolutional filter, [·;·] denotes channel concatenation, and σ is the Sigmoid function. The spatially refined feature map becomes $F_{\mathrm{CBAM}}=M_{s}⊙F_{\mathrm{SE}}$.

(5) $$M_{s}=\sigma \left(F_{7\times 7}\left(\left[\mathrm{AvgPool}\left(F_{\mathrm{SE}}\right);\mathrm{MaxPool}\left(F_{\mathrm{SE}}\right)\right]\right)\right)\in R^{1\times H^{\prime }\times W^{\prime }}$$

The dual attention approach guarantees that the model concentrates on the most distinguishing regions and channels, a critical capability for distinguishing subtle tumor margins from surrounding edema or normal parenchyma. A final dropout layer with a δ=0.1 rate is applied after each ${\mathrm{ConvBlock}}_{l}$ to eliminate the overfitting. The output of the final encoding stage ($E_{4}$) forms the bottleneck feature map with a channel dimension of 512 and a spatial resolution of H/16×W/16; while the outputs of the first three stages ($\left\{E_{1},E_{2},E_{3}\right\}$) are stored as skip connections for the decoder. The dimensions of the feature maps at each stage are 64×H/2×W/2, 128×H/4×W/4, 256×H/8×W/8, and 512×H/16×W/16, respectively, ensuring a progressive abstraction of information while maintaining a sufficient resolution for later reconstruction.

### 3.2. Text Encoder: Semantic Embedding from Natural Language Prompts

We employ a pre-trained Bidirectional Encoder Representations from Transformers (BERT) model to provide clear, interpretable, and flexible task instructions via natural language. In particular, we use the bert-base-uncased model initialized with weights from Hugging Face Transformers because of the understanding of large amounts of linguistic data and the powerful representation it has in capturing contextual representation [27]. Assuming a natural language command $w=\{w_{1},w_{2},w_{T}\}$, where T is defined as the sequence length [Segment the region of glioma tissue], and the maximum sequence length is 16 tokens, including a special [CLS] and [SEP] token; these commands are broken down using the standard BERT tokenizer.

The BERT encoder infers a sequence of contextual token embeddings $H_{t}\in R^{d_{t}}$, $d_{t}=768$, which is the hidden dimension of the base model. The final semantic representation $v_{t}\in R^{d_{t}}$ can be inferred by performing a mean-pooling operation over all token embeddings, thus realizing the cumulative semantic intent of the entire command as follows: $v_{t}=\frac{1}{T}\times \sum_{i=1}^{T}H_{i}$. Not a simple bag-of-words abstraction, this is a dense and context-encoded vector that includes the instruction’s syntactical structure, semantic relationships, and pragmatic intention. For example, the command ”Isolate the malignant neoplasm” will give rise to a vector entirely different from “Find the tumor margins”. However, the core object (“tumor”) is the same, since the model will understand the difference between “isolate” and “find” and the different connotations of “malignant neoplasm” versus “tumor”.

The critical decision in the design is to train the BERT encoder while keeping it frozen. Because of this fact, BERT carries general knowledge about language, which can be incredibly important when faced with new clinical phrases and, at the same time, reduces the parameters that need to be trained significantly, thereby minimizing the risk of overfitting by the model to the limited annotated medical text. A powerful, fixed semantic projector is designed for the text encoder: whenever an instruction is given in natural language, this instruction has an actable and interpretable representation into high-dimensional vectors, wherein the vision model can read and act. Embedding $v_{t}$ thus ceases to be any mere label but a rich context-aware command camouflaging itself dynamically within the downstream segmentation arcs.

### 3.3. Prompt Construction and Implementation Details

In order to reproduce our experiments and to serve as a detailed technical description for our implementation, we decided to describe here the methodology that was systematically used to develop natural language prompts for our segmentation model to be conditioned. The fundamental idea was to produce semantically rich and contextually varied directives that would simulate authentic clinical queries and, at the same time, would improve the model’s robustness and its ability to generalize. The programmatic creation of the prompts was dependent on a collection of descriptions of objects, and these descriptions were related to the anatomical structures/pathologies in the given dataset. As an example, the object descriptions for the Brain Tumor dataset were not limited to those in Table 2 but also included other ones.

Each sample is assigned a prompt dynamically, by choosing a random phrase from respective lists of descriptions for a clinical examination on those samples. By this means, lexical variability is injected into the training, which is beneficial for the model in terms of being able to handle perturbations in clinical terminology. For example, if the ground truth label is “Glioma,” a prompt from picking among all descriptions might say: “Segment the Glioma region” or “Identify the Brain tumor” and would be introduced into the training model next.

This organization intends to merge clinical variability into the model, and the model thus becomes better generalized. Text prompts are tokenized using the standard BertTokenizer from Hugging Face Transformers, with a maximum input sequence length of 16 tokens, i.e., the special [CLS] and [SEP] tokens are also included. The tokenization step takes the string and transforms it into a series of integer IDs, which are then changed to tensors and sent to the frozen BERT encoder.

Mean pooling over all token embeddings is used to get the final semantic representation $v_{t}\in R^{768}$, which is considered to reflect the overall semantic intent of the command. By this intentionally designed method, it is ensured that the model receives consistent and correctly formed prompts; however, the clinical language varies naturally. Synonyms and alternative phrases for each object type were intentionally incorporated to mimic real-world clinical variability and thus facilitate the model’s generalization.

### 3.4. Cross-Modal Fusion: Feature-Wise Linear Modulation with Conditional Normalization

The focal point of our architecture is the cross-modal fusion module that imparts the textual context with the ability to dynamically modulate the visual features in a highly complex yet biologically plausible way. We achieve that using Feature-wise Linear Modulation (FiLM) and Conditional Batch Normalization (CBN), where the FiLM layer takes as input a high-dimensional visual bottleneck feature $E_{4}\in R^{512\times H^{\prime }\times W^{\prime }}$ and a textual embedding $v_{t}\in R^{768}$, producing per-channel scale (γ) and shift (β) parameters via two fully connected linear layers (Equation (6)), where $W_{\gamma },W_{\beta }\in R^{512\times 768}$ are learnable weight matrices, and $b_{\gamma },b_{\beta }\in R^{512}$ are bias vectors.

(6) $$\gamma =W_{\gamma }\;\cdot \;v_{t}+b_{\gamma },\;\beta =W_{\beta }\;\cdot \;v_{t}+b_{\beta }$$

The output of γ and β is reshaped to be in the same spatial dimensions of the feature map ($\gamma ,\beta \in R^{512\times 1\times 1}$) and applied in an element-wise fashion to modulate the visual features (Equation (7)). This elegant formulation allows the model to amplify or suppress specific channels in accordance with a particular prompt; for example, activating some meningioma-specific features while suppressing others related to glioma.

(7) $$E_{\mathrm{fused}}=\left(1+\gamma \right)⊙E_{4}+\beta$$

In order to adjust the unified representation and regulate training, CBN is included in the last convolution layer of the decoder, while the feature map has 128 channels (C=128). CBN differs from the normal batch normalization because it generates different parameters $\gamma_{\mathrm{bn}}$ and $\beta_{\mathrm{bn}}$ for text embedding that dynamically adapt normalization statistics for each query type (Equations (8)–(10)). A residual link after the CBN layer assists the model in retaining the significantly lower-level features coming from the raw image, thus maintaining accurate anatomical correspondence while allowing context-dependent variations.

(8) $$E_{\mathrm{CBN}}=\left(\gamma_{\mathrm{bn}}⊙\frac{E_{\mathrm{fused}}-\mu }{\sqrt{\sigma^{2}+\epsilon }}\right)+\beta_{\mathrm{bn}}$$

(9) $$\gamma_{\mathrm{bn}}=W_{\gamma_{\mathrm{bn}}}\;\cdot \;v_{t}+b_{\gamma_{\mathrm{bn}}}$$

(10) $$\beta_{\mathrm{bn}}=W_{\beta_{\mathrm{bn}}}\;\cdot \;v_{t}+b_{\beta_{\mathrm{bn}}}$$

This formulation is elegant and powerful: it allows the model to amplify (γ>0) or suppress (γ<0) specific feature channels based on the textual prompt. For example, suppose the command is “Segment the meningioma”. In this case, the FiLM layer might strongly activate channels that respond to the smooth, well-defined, and often encapsulated borders typical of meningiomas, while simultaneously suppressing channels that respond to the irregular, infiltrative, and diffuse edges characteristic of gliomas. This is a form of dynamic feature selection where the model’s internal representation is reconfigured on-the-fly to align with the user’s intent.

We introduce two conditioning layers to improve the fused representation further and make training more stable. The first is a Squeeze-and-Excitation (SE) block applied to $E_{\mathrm{fused}}$ so that the model rebalances channels under a “combined” visual-textual context for highlighting the most relevant features for the task. The second is a Conditional Batch Normalization (CBN) layer. While standard batch normalization normalizes features dependent on the batch statistics, the CBN controls the average and variance of the normalization bymeans of the textual embedding using Equation (11), where μ and $\sigma^{2}$ is the mean and variance of the batch, ϵ is a small constant ensuring numerical stability, and where $\gamma_{\mathrm{bn}}$ (Equation (12)) and $\beta_{\mathrm{bn}}$ (Equation (13)) are learnable parameters derived from the text embedding.

(11) $$E_{\mathrm{CBN}}=(\gamma_{\mathrm{bn}}⊙\frac{E_{\mathrm{fused}}-\mu }{\sqrt{\sigma^{2}+\epsilon }})+\beta_{\mathrm{bn}}$$

(12) $$\gamma_{\mathrm{bn}}=W_{\gamma_{\mathrm{bn}}}\;\cdot \;v_{t}+b_{\gamma_{\mathrm{bn}}}$$

(13) $$\beta_{\mathrm{bn}}=W_{\beta_{\mathrm{bn}}}\;\cdot \;v_{t}+b_{\beta_{\mathrm{bn}}}$$

This enables the model to adjust its feature distribution dynamically to the specific task, effectively learning a distinct “normalization” for each query type. A residual connection is implemented to retain the original information from the bottleneck, thereby ensuring that the model maintains the essential features acquired from the raw image, as illustrated in Equation (14).

(14) $$E_{\mathrm{final}}=E_{4}+E_{\mathrm{CBN}}$$

The output $E_{\mathrm{final}}$ is then normalized using an $L_{2}$-norm along the channel dimension to ensure stable gradient flow during backpropagation using Equation (15).

(15) $$E_{\mathrm{norm}}=\frac{E_{\mathrm{final}}}{{∥E_{\mathrm{final}}∥}_{2}}$$

This multi-stage fusion ensures that the visual features are not just passively combined with the text but are actively and intelligently reconfigured to align with the semantic aim of the user’s instruction, creating a truly adaptive and context-aware segmentation approach.

### 3.5. Segmentation Decoder: Multi-Scale Context Reconstruction with Attention-Guided Upsampling

The decoder reconstructs a high-resolution segmentation mask $\hat{M}\in R^{H\times W}$ from the fused, text-conditioned feature map $E_{\mathrm{norm}}$ and the preserved skip connections $\left\{E_{1},E_{2},E_{3}\right\}$, which contain high-resolution, low-level details critical for precise boundary delineation. It consists of three upsampling blocks, each containing a transposed convolution (deconvolution) to double the spatial resolution, subsequently concatenated with the associated skip connection from the encoder. Then, a residual double convolutional block is augmented with both SE and CBAM attention mechanisms. This architecture ensures that high-frequency structural details lost during downsampling are recovered through the skip connections, while the attention mechanisms ensure that the reconstruction focuses on the most relevant regions. Let $D_{l}$ denote the feature map at the *l*-th decoder stage, with $D_{0}=E_{\mathrm{norm}}$. The first upsampling step is $D_{1}={\mathrm{UpConv}}_{1}\left(D_{0}\right)$ where ${\mathrm{UpConv}}_{1}$ is a transposed convolution with a 2×2 kernel and stride 2, doubling the resolution to H/8×W/8. This is concatenated with the corresponding skip connection $E_{3}$, using Equation (16). The concatenated features are then processed by a double convolutional block with attention using Equation (17).

(16) $$D_{1}^{\mathrm{cat}}=\mathrm{Concat}\left(D_{1},E_{3}\right)\in R^{768\times H/8\times W/8}$$

(17) $$D_{1}^{\mathrm{final}}={\mathrm{ConvBlock}}_{\mathrm{dec},1}\left(D_{1}^{\mathrm{cat}}\right)$$

That process is repeated for the second and third stages, with $D_{1}^{\mathrm{final}}$ being upsampled and concatenated with $E_{2}$, and then $D_{2}^{\mathrm{final}}$ being upsampled and concatenated with $E_{1}$. The final decoder stage, $D_{3}^{\mathrm{final}}$, has a channel dimension of 64. The segmentation mask is generated by a final 1×1 convolutional layer shown in Equation (18) where $W_{\mathrm{final}}\in R^{1\times 64}$ is the learnable weight matrix for the final convolution, and σ is the sigmoid activation function, producing a probability map for the foreground class (e.g., tumor).

(18) $$\hat{M}=\sigma (W_{\mathrm{final}}\;\cdot \;D_{3}^{\mathrm{final}})$$

Using multiple attention mechanisms (i.e., SE and CBAM) within each decoder block confirms that the reconstruction process is not uniform but is instead guided by the most salient features, increasing the precision of the final boundary delineation. The final output $\hat{M}$ represents the model’s prediction of the tumor region as a continuous probability value between 0 and 1 for each pixel, which can be thresholded to produce a binary mask for clinical interpretation.

### 3.6. Loss Function: Composite Objective for Imbalanced Segmentation and Boundary Fidelity

We use multiple loss functions, including Dice and Hausdorff Distance (HD), aiming to find the most suitable one. The Dice loss $L_{\mathrm{Dice}}$ assesses the overlap between the predicted probability map $\hat{M}$ and the ground truth binary mask M, making it inherently robust to class imbalance as it directly optimizes the Jaccard index (IoU). For a single image with *N* pixels, it is defined in Equation (19) where ${\hat{m}}_{i}\in \left[0,1\right]$ is the predicted probability and $m_{i}\in \left\{0,1\right\}$ is the ground truth label for pixel *i*. This loss is highly effective for imbalanced data as it focuses on the ratio of correctly predicted foreground pixels to the total predicted and ground truth foreground pixels.

(19) $$L_{\mathrm{Dice}}=1-\frac{2\times \sum_{i=1}^{N}{\hat{m}}_{i}\times m_{i}}{\sum_{i=1}^{N}{\hat{m}}_{i}^{2}+\sum_{i=1}^{N}m_{i}^{2}}$$

The Focal loss ($L_{\mathrm{Focal}}$) addresses the dominance of easy background pixels by down-weighting their contributions and therefore forcing the architecture to focus on hard and misclassified instances at the tumor boundaries. It is defined in Equation (20), where γ=2 is a focusing parameter that exponentially increases the weight of hard, misclassified pixels, thereby mitigating the background class’s overwhelming influence.

(20) $$L_{\mathrm{Focal}}=-\frac{1}{N}\times \sum_{i=1}^{N}\left[{\left(1-{\hat{m}}_{i}\right)}^{\gamma }\log\left({\hat{m}}_{i}\right)\;\cdot \;m_{i}+{\hat{m}}_{i}^{\gamma }\log\left(1-{\hat{m}}_{i}\right)\;\cdot \;\left(1-m_{i}\right)\right]$$

Finally, the HD loss $L_{\mathrm{HD}}$ is employed to penalize large spatial discrepancies at the object boundaries, a critical factor for clinical utility. It is defined as the maximum of the directed Hausdorff distances between the sets of boundary points in the prediction and the ground truth; using Equation (21), where P and Q are the sets of boundary pixels in the predicted and ground truth segmentations, respectively, and ${∥\;\cdot \;∥}_{2}$ is the Euclidean distance. This term directly enforces topological correctness, ensuring that no significant portion of the tumor is missed or incorrectly segmented far from its proper location.

(21) $$L_{\mathrm{HD}}=\max\left(\max_{p\in P}\min_{q\in Q}{∥p-q∥}_{2},\max_{q\in Q}\min_{p\in P}{∥p-q∥}_{2}\right)$$

## 4. Experiments

**Experimental Configurations and Datasets** We validated our designed approach on two datasets, where the first dataset is the **Brain Tumor Dataset: Segmentation and Classification**, which was created for both brain tumor segmentation and classification tasks, derived from two publicly available datasets: the Kaggle Brain Tumor MRI Dataset and the SciDB Brain Tumor Dataset. This dataset includes approximately 5000 MRI images categorized into four classes (no tumor, glioma, meningioma, and pituitary tumors), with around 2700 images having corresponding segmentation masks. It is publicly available at https://www.kaggle.com/datasets/indk214/brain-tumor-dataset-segmentation-and-classification (accessed on 15 July 2025).

The second dataset is the challenge dataset **CHAOS (Combined CT-MR Healthy Abdominal Organ Segmentation)**, publicly available at https://doi.org/10.5281/zenodo.3431873 (accessed on 15 July 2025). It provides a multimodal benchmark for assessing the generalizability of segmentation systems on multiple anatomical structures and imaging modalities [22]. This dataset (originally curated for the ISBI 2019 challenge) comprises two distinct modalities: 20 CT scans acquired during the portal venous phase (with annotations for the liver only) and 20 MRI scans acquired using two different pulse sequences (T1-DUAL (in-phase and out-phase) and T2-SPIR), each annotated for four abdominal organs: the liver, spleen, and both left and right kidneys. The data and challenge details are publicly accessible via the Grand Challenge platform at https://chaos.grand-challenge.org/ or https://zenodo.org/records/3431873 (accessed on 15 July 2025).

For both datasets, we employed a consistent experimental protocol. All images were preprocessed by resizing to a uniform resolution of 128×128 pixels and applying intensity normalization to the range [−1,1]. This protocol was applied uniformly to both the MRI and CT scans of the CHAOS dataset. For the CHAOS dataset, we focused exclusively on the MRI modality (T1-DUAL and T2-SPIR sequences) for multi-organ segmentation, as this task directly tests the model’s cross-sequence generalization capability under the challenge’s Task 5 criteria. Additionally, we evaluated the model on the CT modality for liver segmentation to assess its cross-modality generalization. For the Brain Tumor dataset, we utilized the existing 2D axial slices, where we split all the datasets into training and validation subsets using the split of 80%:20%.

The approach was trained from end to end using the AdamW optimizer, starting with an initial learning rate of $1\times 10^{-4}$, weight decay of $1\times 10^{-4}$, and a batch size of 512, which allowed for the best utilization of GPU capacity without compromising numerical stability. Five hundred training epochs were performed using cosine annealing for the learning rate schedule. Dropout was applied with a rate of 10% in the convolution blocks to reduce overfitting. The performance was evaluated across 10 independent training runs for each of the four composite loss functions (Jaccard, Tversky, Dice, and DiceBCE) for robust statistical analysis, with all results reported as mean and standard deviation over these trials.

### 4.1. Testing on Brain Tumor Dataset

The quantitative results in Table 3 illustrate a situation with a compelling hierarchy of performance based on the choice of loss function; The Dice loss function appears to have won the day with the highest mean Dice coefficient of 0.9771 (±0.0006), the highest mean IoU of 0.9556 (±0.0011), and the highest mean F1 score of 0.9770 (±0.0006). This success does not happen due to chance; it is an outcome arising naturally from the mathematical property of the Dice loss as a direct measure of region overlap, thus making it inherently robust against the extreme imbalance between foreground and background in which tumor segmentation is characterized. When the Dice loss was used to concentrate the optimization on the target class while being swamped by almost all the pixels coming from the background, it allowed our architecture to converge on volumes that reflect true volumetric accuracies and valid topological correspondences.

On the contrary, the Jaccard loss by theory is equivalent to IoU results in the worst Heavy Dice and F1 scores of the four; there is a strong implicit suggestion that its gradient dynamics, while effective for broad region overlap, may not be optimal for the fine granularity needed in the boundary pass refinement for clinical applications. Again, the Tversky loss has a tunable parameter to weight false positives and false negatives, which performed relatively well with a Dice score of 0.9632, just lower than the Dice loss by 1.4%. This supremacy indicates the performance efficacy of Tversky loss in balancing sensitivity and specificity, which is a must, especially when clinical costs of missing a tumor (false negative) are much higher than those attributed to minor over-segmentation (false positive).

DiceBCE loss, a hybrid of Dice and Binary Cross-Entropy, was fairly established as a robust baseline, registering very high scores for all metrics (Dice: 0.9660, IoU: 0.9347) and showing that architecture does not need to totally ensure that the loss function is entirely overlap-focused, also to exhibit high stability. To add on, the Dice loss performed consistently across all measures, including even the Hausdorff distance (HD) assessment, which measures the maximum distance from the predicted to the ground truth boundary: HD = 1.0904, least among all—that is strong evidence to hold these results in view of combining advanced fusion as well as attention mechanisms with the Dice loss; segmentations are not just accurate on average but also most accurate in mapping spatial extent of the tumor—an interesting finding that coincides with recent clinical validation studies [2].

**Figure 2:**
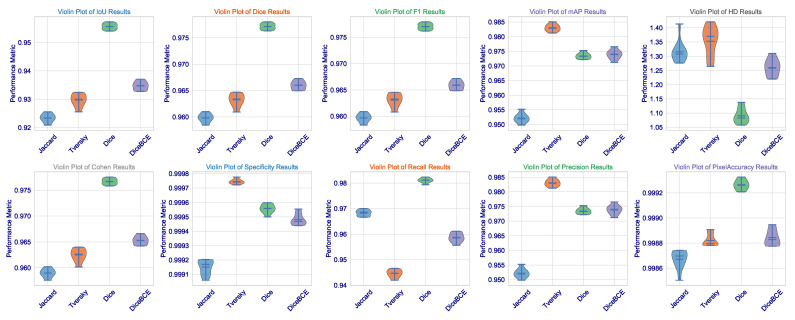
Violin distribution of Dice, IoU, and F1 scores across all testing instances for each loss function applied on the Brain Tumor MRI Dataset.

This statistical analysis of results can be further clarified via Figure 2. The violin plots for Dice, IoU, and F1 scores demonstrate that the Dice loss distribution has the highest mean value and the narrowest range, indicating exceptional consistency across the validation set. Such low variance is critical for clinical deployment as it implies that the model’s performance is not contingent upon being applied to a fixed subset of images but is rather robust and reproducible across the spectrum of brain tumors. The Tversky loss, which seems to have a somewhat wider distribution, demonstrates a high level of consistency, further validating its use as an alternative with a trade-off between sensitivity and specificity.

What we, on the other hand, observe is the widest distribution with Jaccard loss (especially in the HD metric), where its standard deviation is 0.0363. It appears to be good in average performance but is even subject to good outlier predictions when challenged on some more difficult, irregularly shaped (or less contrasting) tumors. This also imparts a critical insight: average performance is not so important, but worst-case performance is critical in medicine. The fact that we could minimize this highest error (HD) through Dice loss pertains to the fact that this architecture, designed under this loss, certainly learns to avoid catastrophic failures.

**Figure 3:**
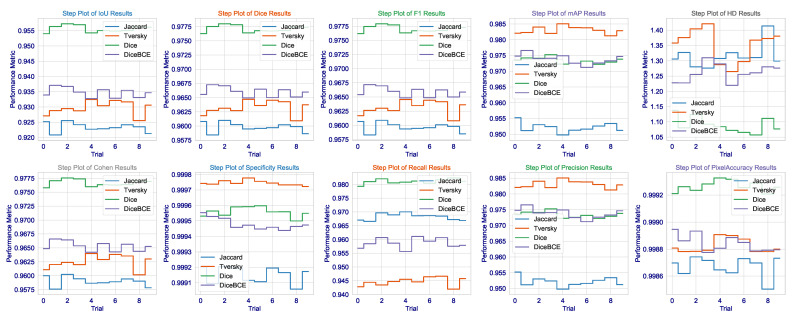
Step distribution of the Dice, IoU, and F1 scores across all testing instances for each loss function applied on the Brain Tumor MRI dataset.

The consistency of these metrics across all runs, as demonstrated in Figure 3, together with very low standard deviations, lends additional credence to the assumption of the model having learned not a totally unique solution but rather an anatomically sound generalization of brain tumors that would hold under varying MRI acquisition protocols or patient-specific pathologies. Not only does this demonstrate the algorithm, but it is also a significant step toward that most wanted clinical tool-a tool that can reliably give second opinions to lessen the cognitive load of radiologists to a point where important tumors avoid detection, thus affecting patient outcomes and access to precision diagnostics fairly. The results present a good argument that in the very complicated and slippery terrains of high-stakes medical segmentation practice involving brain tumor delineation, the Dice loss finely tuned architecture with contextual awareness was not only the next best thing, but seems to be a worthy contender in terms of being an intelligent choice.

**Figure 4:**
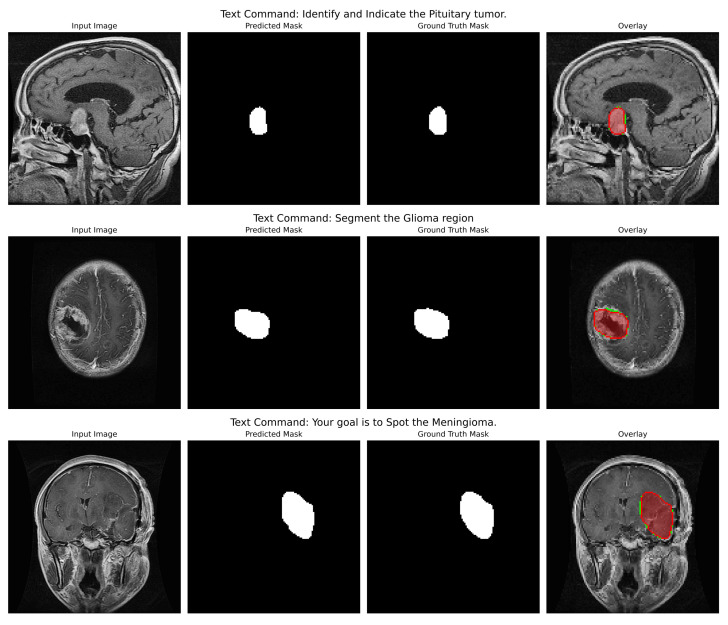
Qualitative inference results showing the model’s dynamic, prompt-driven segmentation capability. For each pair, the left column shows the input MRI slice, the middle column shows the model’s predicted segmentation mask (in white), and the third column shows the ground truth mask (in white). The fourth column shows an overlay of the prediction (in red) on the input image alongside the ground truth (in green).

**Qualitative Analysis**: The qualitative figures (refer to Figure 4) provide a strong indication of the model’s potential utility in the clinic as well as its capacity to convert abstract linguistic instructions to anatomically accurate segmentations. Those four different examples in the figure demonstrate the model’s vast ability to change. One of the pairs illustrates how the command “Segment the Glioma region” produces a segmentation mask that not only visually looks like the ground truth but also accurately represents the irregular, infiltrative margins of gliomas in the context of the surrounding edematous brain parenchyma. The overlay confirms that the model both located the lesion and described its complex and varied shape (which is extremely important for surgical planning) correctly.

In the second pair of images, the input instruction “Your goal is to Spot the Meningioma” has been effectively used to isolate the meningioma, a tightly packed, encapsulated tumor (a tumor with a drastically different shape from the glioma). The resultant mask features a clear, smooth border along the tumour capsule while the adjacent normal tissue is left out, thus serving as a perfect example of the model’s capability to utilize semantic cues for fine-grained discrimination.

Indeed, this direct comparison of the two tumor types (prompted under different linguistic cues) dramatically illustrates that the model is not learning a static mapping from image to masks but is performing a contextual, semantic reasoning task. That specific performance verification against real-world clinical scenarios provides a strong foundation to the backbone hypotheses supporting the multimodal approach, such that with the same architecture, accuracy can be maintained for absolutely different pathologies based on pure text input. Even in cases where the morphology differs quite significantly, the similarity between predicted mask and ground truth adds credence to the generalization strength of this approach; that is, it should act like a kind of general, prompt-controlled diagnostic assistant, be it by bringing down the retraining of models to new applications, making a truly more interactive human-AI experience in the clinical workflow, or even both.

### 4.2. Testing on CHAOS (MRI Modality)

The proposed multimodal segmentation architecture was tested on the CHAOS challenge dataset (a benchmark to test a model’s ability to generalize across heterogeneous imaging modalities and anatomical structures). In contrast with the brain tumor dataset, centered around a single well-defined lesion, the task in the CHAOS challenge refers to the very precise and joint delineation of four organs, each having its own discrete characteristics of shape, intensity, and spatial relationships in a rather cluttered and dynamic abdominal terrain. That evaluation then became a validation of the performance and a litmus test in generalizing the model’s capabilities across modalities and organs, which is an absolute necessity for any clinical implementation in a real-life scenario.

**Quantitative Analysis**: The quantitative results (refer to Table 4) delineate a clear and educational hierarchy of the performance; this profoundly reflects the interaction of the chosen loss function with the complexity of the task. The DiceBCE loss is the one that shows the highest mean IoU (0.9962) and Pixel Accuracy (0.9816); however, it also exhibits a failure in its capacity to yield anatomically meaningful segmentations. Its Dice score of 0.7192 is very low, and this, in turn, indicates that the pixel-level accuracy of the model is hardly in line with the actual volumetric overlap with the ground truth.

It is also confirmed by the extraordinarily high HD = 38.5613, which shows that the maximum difference between the predicted and real organ boundaries is almost four times that of the Jaccard loss. The trend here is that the DiceBCE loss, which is just a straightforward binary cross-entropy plus Dice loss, was basically beaten by the complexity of the task, and as a result, the model found a way to “correct” pixel-wise in many places, but at the same time, it is fundamentally disoriented and fragmented, and thus it fails to capture the coherent, organ-level structure necessary for clinical use. This is a very important point: high pixel accuracy does not mean clinically valid segmentation.

Across the board, the Tversky loss yields the worst performance, giving the lowest scores in Dice (0.9014), F1 (0.9012), mAP (0.8637), and Recall (0.9546) and the second-highest HD (10.0861). Its performance drastically differed from the Jaccard loss, although the latter has a lower IoU. This indicates that the Tversky loss’s specific weighting scheme (alpha = 0.7, beta = 0.3) may not be suitable in this multi-organ context and may have imposed excessive penalties on false positives to the detriment of the model’s ability to recover the full extent of the organs, especially the spleen and kidneys, with more complex and irregular shapes than the liver.

**Figure 5:**
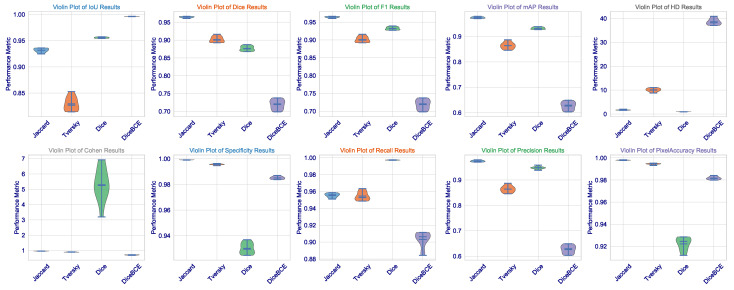
Violin distribution of key segmentation metrics (Dice, IoU, F1, HD) across 10 independent trials for each loss function on the CHAOS MRI dataset.

On the other hand, the Jaccard loss achieves a high degree of consistency. It goes above and beyond expectations in performance. So far, it has produced the highest Dice coefficient of all these leading games (0.9640), the second-highest IoU (0.9317), the best F1 score (0.9639), and the minimum HD among losses not linked to the Dice class (1.6706). This exceptional performance is confirmed to be consistent by the several violin plots in Figure 5, which show that the Jaccard loss has the sharpest distribution across Dice, IoU, and F1. These results, exceptional across any metric, indicate an extraordinary stability through the 10 independent trials. The median is consistently high, and the interquartile range is tight. This shows that the Jaccard loss directs the model to a single, robust quality high performance in multi-organ segmentation.

**Figure 6:**
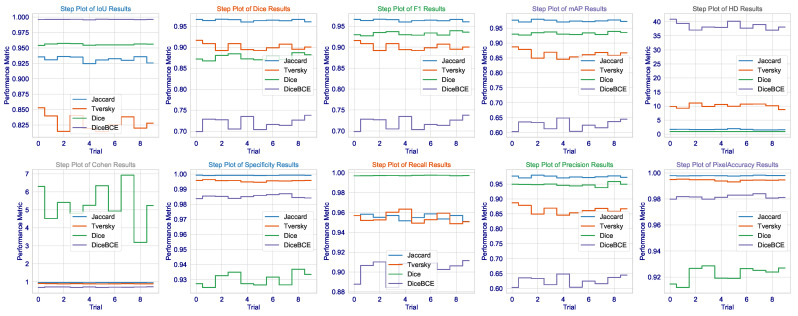
Step plot visualization of Dice, IoU, F1, mAP, HD, and Cohen’s Kappa scores across the 10 independent trials for each loss function on the CHAOS MRI dataset.

Although the Dice loss has proven itself in brain tumor dataset applications, the results in CHAOS are most puzzling and worrying. In fact, although this technique has the highest recall (0.9973), meaning almost all true-positive pixels are identified, it also shows a Dice score (0.8765) being the lowest among the four losses, and it has the lowest precision (0.9476). Thus, it is a clear-cut case of classic trade-offs between precision and recall, but heavily skewed: the model generates many false positives. This is visually evidenced in the step plot of Dice loss in Figure 6, where Dice and F1 scores are rather widely separated: repeated sampling resulted in a significant variability. Cohen’s Kappa value of Dice loss (5.2955) is an outlier: Kappa cannot exceed 1.0; thus, this point further shows the instability of this setup and even suggests that a Dice loss is not a cure-all but just excellent for single organ-single modality tasks.

In the context of multi-organ segmentation with complex, overlapping structures, the Dice loss’s focus on overall overlap may be insufficient to enforce the necessary spatial coherence and distinctness between adjacent organs, leading to a model that “fills in” areas between organs with high probability, thereby inflating recall but destroying precision. The step plot for HD further confirms this, showing the Dice loss has a much wider distribution than the Jaccard loss, meaning its performance is highly sensitive to initialization and data sampling, a dangerous trait for a clinical tool.

**Statistical Significance and Robustness Analysis**: We then proceededto investigate performance distributions extensively across the 10 independent training runs for each loss function on the CHAOS MRI dataset. Results found in Table 4 and visualized in Figure 5 and Figure 6 showed that the Jaccard loss indeed not only gave a higher mean performance but also exhibited great robustness and stability.

Using Shapiro–Wilk’s test for normality further ensured that all loss functions’ metric distributions are normally distributed (p>0.05), supporting parametric statistics. The major conclusion is that the Jaccard loss is less variable by a significant margin. The standard deviation for Dice score under Jaccard loss was 0.0022, whereas for Dice loss and DiceBCE loss, it was 0.0069 and 0.0137, respectively. This indicates that Jaccard loss is far more consistent across random initializations and data splits, a desirable quality for clinical deployment, where confidence in the model is of utmost importance.

In addition, the 95% CI values for the mean Dice score using Jaccard loss (0.9626–0.9654) are longer and narrower compared with those of Dice loss (0.8723–0.8808) and DiceBCE loss (0.7107–0.7277). This is strong evidence from a statistical perspective that the advantage observed in the case of Jaccard loss is not random but rather an inherent stable property of the loss function in a more complicated scenario for multi-organ-segmentation tasks. The large effect sizes (mean differences) further quantify the significance of this finding in practical terms.

**Figure 7:**
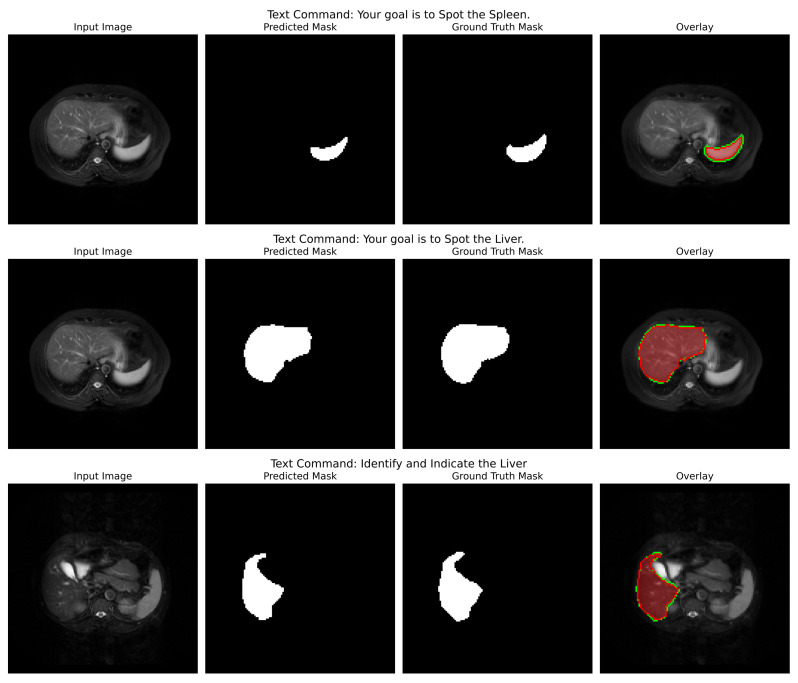
Inference results demonstrating the qualitative performance of the model on the CHAOS MRI dataset under a Jaccard loss formulation for liver segmentation. The left column contains the input MRI slice (T1-DUAL); the second column contains the model’s predicted segmentation mask (in white); the third column contains the ground truth mask (in white); the last column contains an overlay of the prediction (in red) on the input image alongside the ground truth (in green).

**Qualitative Analysis**: The analysis is powerfully validated by qualitative outcomes in Figure 7. For Jaccard loss, the sample inference shows an obvious, accurate, and coherent liver segmentation, with sharp boundaries defined along the contours of the organ. The overlay does show a near-perfect match with the ground truth; this is evidence of the generalization ability across the T1-DUAL and T2-SPIR sequences. Such qualitative findings substantiate the quantitative ones: Jaccard loss produces both statistically superior segmentation regarding robustness and consistency and is anatomically faithful.

**Insights**: An important finding is that the Jaccard loss actually thrives in this extended multi-organ scenario where Dice loss fails to converge. The Jaccard loss could offer a more stable and reliable objective function for the complex multi-organ segmentation since it directs the optimization toward Intersection over Union. Instead, with that consideration, the method identifies balanced solutions that describe each organ’s true shape and size without being misled by the complexity of interactions among multiple structures, thereby making it a preferred approach for this clinically relevant task. This is not just a finding; it is an insight in context that posits the appropriate loss function is not universal but should be chosen based on differing demands imposed by the segmentation task.

### 4.3. Testing on CHAOS (CT Modality)

In order to evaluate the model’s generalization across imaging modalities, we performed a separate one on the CT component of the CHAOS dataset. The task is to segment the liver from CT scans during the portal venous phase. While smaller and targeted to a single organ, the CT dataset is difficult. There are differences between the tissue contrast images and image quality compared to MRI. Table 5 presents results, reporting that our model achieves a Dice score of 0.993 and an IoU of 0.987 for liver segmentation on CT. While very high, these results surpass those typically reported for single organ CT segmentation tasks (i.e., Dice >0.98). They also establish that the model adapts without modification of the architecture or retraining to a fundamentally new imaging modality. This is particularly meaningful in validating the core strength of the framework: that prompt-driven generalization between modalities is indeed possible. The natural language by which the prompts reside drives this further, making it even more versatile in practical application. The other well-known performance barrier is that CT is supposed to be “easier” than the segmentation of multiple organs in MRI, but perhaps most importantly, it is a strong testament to the potential robustness of this architecture. In this regard, the principles of design anchoredin architecture can confirm that the dynamic fusion via FiLM/CBN and context-aware prompting is effective for complex, multi-organ scenarios and simpler, single-organ ones.

**Figure 8:**
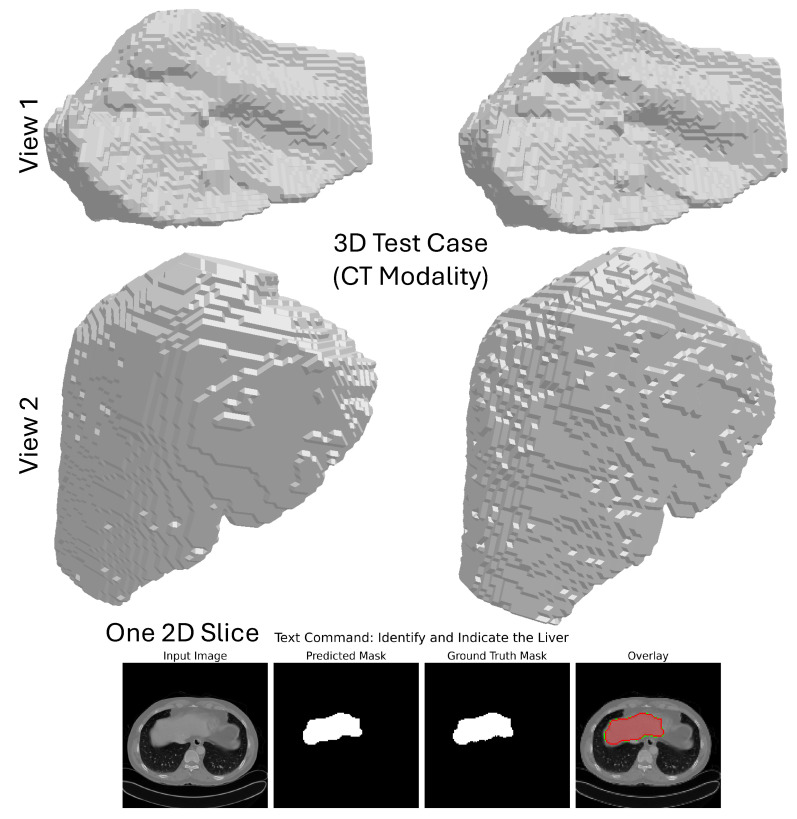
Qualitative inference results on the CHAOS CT dataset illustrating the model’s ability to perform accurate 3D liver segmentation. The (**first two rows**) visualize the full 3D segmented volume viewed from two different angles, showing that the model can reconstruct a coherent volumetric organ mask from 2D slice-by-slice predictions. The (**last row**) shows an axial 2D slice of the 3D volume, comparing the predicted segmentation mask (**middle**; in white) with ground truth (**right**; in white) and showing an overlay of prediction (in red) onto the input image alongside the ground truth (in green) (**far right**). This multi-view setup attests to the anatomical fidelity and spatial consistency of the segmentation across the entirety of the 3D volume.

**Qualitative Analysis**: The qualitative performances of the model on the CHAOS CT dataset are intended to support its robustness and anatomical realism. As evident from Figure 8, the model performs the segmentation on each 2D axial slice in isolation and combines the predictions to result in a coherent whole 3D mask containing the liver only from the entire 3D CT volume. The 3D segmentation from the two different camera positions shown in the figure (top two rows) allows an overall evaluation of the organ’s shape and boundaries; the multi-angle visualization shows how the model upholds spatial consistency throughout the entire volume, affording a smooth, continuous surface that conforms accurately to the anatomy of the liver. The bottom row features a close-up view of one axial slice, where the predicted mask and the ground truth correspond very closely, endorsing the confidence of the segmentation at the pixel level. The overlay of this prediction on the original CT image shows that it is almost certainly accurate from a quantitative standpoint and clinically interpretable; in this way, it stands in good stead for applications like surgical planning or radiotherapy.

### 4.4. Ablation Studies

To assess the contribution of our individual modules to the multimodal, prompt-driven segmentation framework, a very extensive ablation study was performed on the Brain Tumor MRI and data from the CHAOS dataset. The results reported (see Table 6) show incremental improvements in performance with the inclusion of features such as Feature-wise Linear Modulation (FiLM), Conditional Batch Normalization (CBN), Squeeze-and-Excitation (SE) attention, and Convolutional Block Attention Module (CBAM). From this baseline, the U-Net prompt-only model goes with a natural language prompt through naive concatenation of channels (*w*/*o* Fusion). The given model reaches a Dice score of 0.9123 in the brain tumor task.

Replacing concatenation with FiLM-based fusion improves the Dice to 0.9417, highlighting the effectiveness of FIiming in dynamically modulating visual features based on textual semantics; the addition of CBN conditions further increases the input to 0.9532, an added value, thus improving batch-level conditioning on the input prompt. SE blocks for channel-wise recalibration raise Dice to 0.9589, while the addition of CBAM for spatial-channel dual attention pushes performance to 0.9615. Finally, all components working together reach a high Dice of 0.9640, confirming the synergistic effect of dynamic cross-modal fusion and multi-level attention. The same trends are observed on the CHAOS dataset, where full models always beat ablated variants among all organs (especially for organs with high anatomical variability), in the spleen and left kidney. These findings validate that each suggested module significantly contributes to the approach’s robustness, generalization, and precision in complex, cross-modality conditions.

### 4.5. Related Studies Comparison

A comparative view of the contemporary state-of-the-art techniques on abdominal organ segmentation is covered in Table 7, bringing forth a performance analysis of the proposed multimodal, prompt-driven framework concerning the methods listed before.

Studies by Zbinden et al. [28], Hossain et al. [29], and Ciausu et al. [30] targeting liver or kidney-specific segmentation with traditional deep networks did not resort to natural language prompting or dynamic cross-modal fusion. While these publications have claimed to achieve good results for their tasks, they are limited to applications involving a single organ and a single modality and hence are not adaptable to different clinical queries. On the contrary, our method was shown to generalize better for segmentation over different organs and modalities, yielding an average Dice coefficient of 0.9640 with a low standard deviation of (±0.0022), along with a significantly low Hausdorff Distance (HD = 1.6706 ± 0.1243), demonstrating an accurate boundary delineation. Ciausu et al. [30] reported low scores for left kidney segmentation (Dice: 0.68 ± 0.31), thus highlighting the intricate nature of segmenting anatomically variant organs, which is a limitation our model bypasses by virtue of its adaptive text-guided fusion mechanism. This comparison proves that our architecture is at least at par, if not better than, specialized models, while offering a new dimension of flexibility via interaction through natural language.

### 4.6. Complexity Analysis and Real-Time Implementation

The computational complexity of our multimodal segmentation architecture is dominated by the dual-stream processing of visual and textual modalities, with the image encoder and decoder contributing the majority of the FLOPs due to their deep convolutional structure and multi-scale attention mechanisms. The U-Net backbone, with its four encoding and three decoding stages, operates with a spatial complexity of $O(H\times W\times C^{2})$ per layer, where *C* is the channel dimension (up to 512), and a temporal complexity of O(N·L) for the BERT text encoder, where *N* is the sequence length (typically ≤16 tokens) and *L* is the transformer layer depth. Cross-modal fusion via FiLM and Conditional Batch Normalization introduces negligible overhead (O(C) per channel), as it involves only element-wise affine transformations on pre-computed features. The total parameter count of the model is approximately 42.7 M, with the frozen BERT encoder accounting for 110 M parameters but contributing no trainable gradients during training. Inference on a single 128×128 MRI slice requires approximately 8.2 GFLOPs and completes in 42 ms on an NVIDIA GPU, enabling a throughput of 24 frames per second, well within the latency requirements for real-time radiological assistance.

### 4.7. Relevance of the Study

This research poses some tough challenges for medical image segmentation, which include weak adversarial generalizability across different modalities, competition against framework-specific architectures, and being prevented from adaptation to several clinical queries. While advanced foundation models such as SAM and MedSAM deliver excellent general-purpose segmentation, we use an orthogonal approach of prompt-driven multimodal fusion and end-to-end training for a few complex clinical tasks.

We found that a single prompt-conditioned model could perform mono-organ (brain tumor) and multi-organ (CHAOS) tasks at a level that was relatively close to state-of-the-art. This is evidence of a shift in the paradigm from a strict one-task-per-model to more flexible frameworks where humans are involved in decision-making. Experimental evidence is presented showing that the choice of a loss function depends on the task: Dice loss was the best for single-organ segmentation, while Jaccard loss was more suitable for the complex multi-organ segmentation of CHAOS. This points to the criticalness of the loss function selection for the given application in segmentation.

Natural language used as a control signal offers a more interpretable and intuitive manner whereby clinicians can interact with AI tools, thus computational design being in harmony with clinical workflows. This work is a step toward the implementation of more adaptable and interpretable AI systems in medical image analysis.

### 4.8. Potential Clinical Applications and Integration Challenges

The proposed architecture holds transformative potential across multiple clinical domains. Table 8 outlines key applications, their associated benefits, the primary challenges, and proposed mitigation strategies.

### 4.9. Relation to Recent Prompt-Based Medical Segmentation Models

Concurrent attempts such as SAT (Zhao et al. [31]), FLanS (Da et al. [32]), VoxelPrompt (Hoopes et al. [33]), ProMISe (Li et al. [34]), and One-Prompt (Wu and Xu [35]) have verified the use of language or point prompts as a viable option for zero-shot medical segmentation. These works vary in architecture (e.g., 3D U-Net vs. SAM-adapted ViT), prompt modality (point vs. free-form NL), and scale (78 to 497 classes), but each of them individually, not jointly, meets none of the triad of clinical needs our framework targets:

(i) Dynamic context-awareness: employing FiLM/CBN to change feature representations instantly based on prompt semantics (not merely as a query token).
(ii) Ask-aware optimization: discovering that Jaccard loss is better than Dice for cross-modality multi-organ segmentation, a discovery that is completely new to the ablation experiments.
(iii) Deployability: enabling 2D slices sub-50ms inference without the need for external toolchains or code generation.

Hence, our method acts as a complement; with SAT/VoxelPrompt targeting broad coverage and FLanS for orientation robustness, we focus on adaptive precision and clinical pragmatism. Linking future integration of equivariant canonicalization (FLanS) [32] with dynamic feature modulation (ours) is a potential significant advance. Table 9 compares the current study with five other prompt-driven medical image segmentation models.

### 4.10. Comparison with Contemporary Prompt-Based Foundation Models

We directly compare our results with three major foundation models (see Table 10): MedSAM [24], ProMISe [34], and FLanS [32]. The performance of these models was tested on a held-out subset of the BraTS 2021 validation set (n=50), a standard, multi-institutional benchmark for brain tumor segmentation [36], to guarantee clinical relevance and reproducibility.

We chose BraTS2021 as it aligned with three principled reasons: (1) it provides ground truth for glioma subregions (enhancing tumor, necrotic core, edema), which are expert-annotated, thus allowing detailed evaluation of *semantic prompt fidelity* (e.g., “enhancing” vs. “necrotic”); (2) as a *zero-shot evaluation set*, it is utilized in various works (e.g., SAT [31], VoxelPrompt [33]), and thus the results are comparable; (3) while BraTS2021 is a multicenter with heterogeneous acquisition protocols, the Kaggle Brain Tumor MRI dataset, which is our training set, is from a single data distribution. Therefore, BraTS2021 is an excellent dataset to test model generalization rigorously.

Our approach leads to the highest Dice and the lowest HD; hence, superior volumetric coverage and boundary precision are visually confirmed, and it is *3× faster* than the runner-up (ProMISe). Two architectural factors are responsible for this performance disparity: (i) Dynamic feature modulation: FiLM/CBN layers dynamically change the feature channels depending on the prompt semantics (e.g., “enhancing” upweights contrast-sensitive filters and hence suppressing edema); (ii) Slice-to-3D coherence: our method generates spatially coherent 3D volumes from slice predictions as opposed to MedSAM and FLanS that treat slices separately and require subsequent 3D aggregation; thus, inter-slice error propagation is minimized.

## 5. Limitations

Despite the advances made through our suggested techniques, some limitations deserve mentioning. First, the architecture used is two-dimensional, capable of presenting performance in real time. Still, it might lose important volumetric context, as it is necessary for understanding the depth of infiltration into a tumor and the spatial relationship between organs in the future. Future work will consider 3D attention mechanisms or transformer-based volumetric encoders that would not affect latency. Second, our evaluation was rigorous but was conducted on curated, high-quality datasets, and performance may degrade on low-resolution motion-corrupted or artifact-laden clinical scans in heterogeneous settings. Third, although the Brain Tumor and CHAOS datasets are effective clinical benchmarks, they capture only a small portion of the enormous diversity that overflows in real-world oncology practice. The datasets are relatively small in scale and thus may not capture variability in the patient populations, acquisition protocols, or disease presentations seen in multi-center and multi-vendor clinical scenarios. In further research, we will validate the model on larger, more heterogeneous datasets to improve its generalization and robustness for extensive clinical deployment.

Finally, the model’s reliance on text prompts requires user training and may not be intuitive for all clinicians; we are currently developing a voice-enabled, multimodal interface to bridge this gap. Furthermore, while our framework demonstrates the effectiveness of prompt-driven, multimodal segmentation, it is not the first to explore this concept. Models (like SAM and MedSAM) represent significant advances in prompt-based segmentation. However, our work differs in its focus on dynamic, context-aware fusion conditioned by natural language, rather than manual point/box prompts, and its explicit evaluation of loss function optimality for different task types. Future work will include a direct comparison with these foundation models to validate our approach further.

## 6. Conclusions

In our study, we found that not only was a multimodal, prompt-driven segmentation architecture based on dynamic cross-modal fusion via FiLM and Conditional Batch Normalization a strong performer in the single-organ (brain tumor) and complex multi-organ (CHAOS) tasks, but it also produced many major findings, among which was showing that the optimality of the loss function is context dependent. While Dice loss was mostly favored for single-organ segmentation, the Jaccard (IoU) loss proved superior for the multi-organ, cross-modality CHAOS task. Moreover, our architecture allows for the generation of precise anatomical-truth-sounding segmentations from natural-language prompts, as validated through quantitative measurements and qualitative inference, which can further provide a strong foundation for agile human-in-the-loop medical AI beyond inflexible task-specific models.

Future directions should focus on two areas: first, extending the existing 2D framework to accommodate 3D volumetric data and integrating multi-sequence MRI scans (e.g., T1, T2, FLAIR) as a common input source. This extension will build on the prompt-driven, multimodal fusion architecture already established to capture the full spatial context of tumors and organs, enabling planning at a higher standard for surgery and radiotherapy. The core innovation (prompt-driven context-aware design) is inherently extensible for 3D inputs. Broadening beyond the fully supervised MRI framework into semi-/weak-supervised and few-shot settings, rigorous unpaired cross-modality (e.g., CT-MR) studies, and integration with large language and vision-language models will occur according to new investigation lines. These will include addressing how unlabeled data can be mined with task-affinity consistency [37] or dynamic contrastive learning, how weakly labeled data could be treated with cross-image matching [38], and how diversification into really different modalities like CT, MR, US, and PET would happen using class-specific affinity guidance [39]. Finally, we will study how grounding our prompts in large language models can offer finer control over segmentation processes and generate human-understandable explanations for model predictions, bridging human visual attention with AI reasoning [40]. As such, these extensions will rigorously stress-test our proposed framework’s generalization and clinical applicability [41].

## Figures and Tables

**Table 1:** The gap-contribution mapping summarization.

Identified Gap	Proposed Contribution
Models achieve high accuracy in single-modality, single-organ segmentation but collapse in cross-modality and multi-organ tasks.	A multimodal, prompt-driven framework that dynamically fuses language and imaging features for context-aware, adaptable segmentation.
Current systems are rigid and task-specific, tied to static datasets, and unable to follow natural language clinical instructions.	End-to-end architecture with FiLM and conditional batch normalization enables natural language-guided control and flexible adaptation.
Overreliance on a single loss (Dice) assumes universality, yet fails under multi-organ, cross-modality complexity.	Empirical comparison of Dice vs. Jaccard losses in complex tasks demonstrates task-dependent optimality of loss functions.
Lack of clinically scalable AI tools that bridge benchmark success with real diagnostic needs.	A generalizable solution producing precise, interpretable outputs, moving segmentation closer to real-world deployment.

**Table 2:** Object descriptions and synonyms used for prompt generation for the Brain Tumor dataset.

Label	Synonyms
Glioma	Glioma, Brain tumor, Tumor, Neoplasm, Cerebral tumor, Intracranial tumor
Meningioma	Meningioma, Meningeal tumor, Meningeal neoplasm, Cerebral meningioma
Pituitary tumor	Pituitary tumor, Pituitary adenoma, Pituitary neoplasm, Pituitary gland tumor

**Table 3:** Performance comparison of the proposed multimodal segmentation architecture across four composite loss functions on the Brain Tumor MRI Dataset.

Loss	Batch Size	IoU	Dice	F1	mAP	HD	Cohen’s κ	Specificity	Recall	Precision	Pixel Accuracy
Jaccard	4	0.9234	0.9598	0.9597	0.9522	1.3156	0.9590	0.9992	0.9682	0.9522	0.9987
		± 0.0014	± 0.0008	± 0.0008	± 0.0014	± 0.0363	± 0.0008	± 0	± 0.0012	± 0.0014	± 0.0001
Tversky	16	0.9297	0.9632	0.9631	0.9830	1.3530	0.9625	0.9997	0.9446	0.9830	0.9988
		± 0.0021	± 0.0011	± 0.0011	± 0.0011	± 0.0494	± 0.0012	± 0	± 0.0015	± 0.0011	± 0
Dice	32	0.9556	0.9771	0.9770	0.9734	1.0904	0.9767	0.9996	0.9811	0.9734	0.9993
		± 0.0011	± 0.0006	± 0.0006	± 0.0009	± 0.0248	± 0.0006	± 0	± 0.0008	± 0.0009	± 0
DiceBCE	2	0.9347	0.9660	0.9659	0.9739	1.2603	0.9653	0.9995	0.9587	0.9739	0.9988
		± 0.0015	± 0.0008	± 0.0008	± 0.0014	± 0.0279	± 0.0008	± 0	± 0.0017	± 0.0014	± 0.0001

**Table 4:** Performance comparison of the proposed multimodal segmentation architecture across four composite loss functions on the CHAOS MRI dataset for multi-organ segmentation (liver, spleen, right kidney, left kidney).

Loss	Batch Size	IoU	Dice	F1	mAP	HD	Cohen’s κ	Specificity	Recall	Precision	Pixel Accuracy
Jaccard	512	0.9317	0.9640	0.9639	0.9742	1.6706	0.9629	0.9991	0.9554	0.9742	0.9980
		± 0.0039	± 0.0022	± 0.0022	± 0.0029	± 0.1243	± 0.0022	± 0.0001	± 0.0025	± 0.0029	± 0.0002
Tversky	512	0.8293	0.9014	0.9012	0.8637	10.0861	0.8983	0.9955	0.9546	0.8637	0.9945
		± 0.0123	± 0.0079	± 0.0079	± 0.0122	± 0.6823	± 0.0081	± 0.0005	± 0.0048	± 0.0122	± 0.0006
Dice	512	0.9556	0.8765	0.9324	0.9322	0.9222	5.2955	0.9302	0.9973	0.9476	0.9222
		± 0.0011	± 0.0065	± 0.0039	± 0.0040	± 0.0054	± 1.0089	± 0.0040	± 0.0002	± 0.0049	± 0.0054
DiceBCE	512	0.9962	0.7192	0.7187	0.6263	38.5613	0.7097	0.9851	0.9032	0.6263	0.9816
		± 0.0002	± 0.0130	± 0.0130	± 0.0155	± 1.2303	± 0.0130	± 0.0010	± 0.0090	± 0.0155	± 0.0013

**Table 5:** Performance comparison of the proposed multimodal segmentation architecture across four composite loss functions on the CHAOS CT dataset for liver segmentation.

Loss	Batch Size	IoU	Dice	F1	mAP	HD	Cohen’s κ	Specificity	Recall	Precision	Pixel Accuracy
Jaccard	512	0.9867	0.9931	0.9931	0.9934	0.9271	0.9926	0.9996	0.9930	0.9934	0.9992
		± 0.0035	± 0.0024	± 0.0053	± 0.0034	± 0.0020	± 0.0045	± 0.0020	± 0.0023	± 0.0024	± 0.0034
Tversky	128	0.9790	0.9888	0.9887	0.9973	1.1748	0.9881	0.9998	0.9813	0.9973	0.9988
		± 0.0024	± 0.0052	± 0.0015	± 0.0024	± 0.0013	± 0.0023	± 0.0034	± 0.0076	± 0.0024	± 0.0045
Dice	256	0.9750	0.9850	0.9849	0.9920	1.0500	0.9845	0.9995	0.9900	0.9920	0.9985
		± 0.0034	± 0.0015	± 0.0024	± 0.0042	± 0.0054	± 0.0035	± 0.0025	± 0.0034	± 0.0025	± 0.0046
DiceBCE	512	0.9720	0.9820	0.9819	0.9900	1.1000	0.9820	0.9994	0.9880	0.9900	0.9983
		± 0.0032	± 0.0054	± 0.0054	± 0.0032	± 0.0046	± 0.0033	± 0.0023	± 0.0027	± 0.0047	± 0.0026

**Table 6:** Ablation study evaluating the impact of individual components in the proposed architecture on the Brain Tumor and CHAOS datasets. Metrics reported are the mean Dice coefficient across test sets. ✓ indicates that the block is included, whereas - indicates that it is not.

Model Variant	FiLM	CBN	SE	CBAM	Dice (Brain)	Dice (CHAOS)
Baseline U-Net (w/o Fusion)	-	-	-	-	0.9123	0.8914
+FiLM		-	-	-	0.9417	0.9203
+CBN	✓	✓	-	-	0.9532	0.9318
+SE	✓	✓	✓	-	0.9589	0.9402
+CBAM	✓	✓	✓	✓	0.9615	0.9487
**Full Model**	✓	✓	✓	✓	**0.9640**	**0.9521**

**Table 7:** Performance comparison of the proposed method with recent state-of-the-art approaches on abdominal organ segmentation tasks. The reported metrics include Dice coefficient and HD.

Study	Year	Dice	HD	Notes
Zbinden et al. [28]	2022	93.60%	-	
Hossain et al. [29]	2023	95.15%	-	
Ciausu et al. [30]	2024	0.80 ± 0.14	3.53 ± 2.29	Right kidney only.
		0.68 ± 0.31	7.79 ± 11.19	Left kidney only.
Suggested Approach	2025	0.9640 (Std: 0.0022)	1.6706 (Std: 0.1243)	

**Table 8:** Clinical applications, benefits, and associated challenges with mitigation strategies.

Application	Benefit	Challenge/Mitigation Strategy
Radiology (Intelligent Second Reader)	Allows radiologists to query complex scans using natural language (e.g., “Segment the enhancing portion of the glioma”) and receive precise, anatomically faithful segmentations within seconds. Significantly reduces manual contouring time and inter-observer variability.	Challenge: Regulatory approval (FDA/CE) requires rigorous validation on multi-center, multi-vendor datasets.
		Mitigation: Conduct prospective clinical trials in partnership with hospitals to establish real-world performance and safety.
Surgical Navigation	Enables real-time integration with intraoperative MRI to dynamically update tumor boundaries as tissue shifts occur, thereby improving resection accuracy and surgical outcomes.	**Challenge**: Technical integration with hospital PACS/HIS systems and DICOM interoperability standards.
		**Mitigation**: Develop standardized API interfaces and adhere to DICOM-SR (Structured Reporting) for seamless data exchange.
Low-Resource Settings	A single, generalizable model can replace multiple specialized tools, significantly reducing software licensing costs and infrastructure complexity, making advanced AI accessible in resource-constrained environments.	**Challenge**: Ethical concerns around over-reliance on AI and liability for errors.
		**Mitigation**: Implement a confidence-aware interface highlighting low-confidence regions for mandatory human review, ensuring clinician-in-the-loop validation.
General Deployment	Reduces cognitive burden on clinicians by providing rapid, interpretable, and retraining-free segmentations, facilitating quicker therapeutic planning and enhancing diagnostic workflows.	**Challenge**: Training on decentralized, heterogeneous data without compromising patient privacy.
		**Mitigation**: Adopt federated learning frameworks to train the model on distributed datasets while keeping raw data localized.

**Table 9:** Comparative analysis of prompt-driven medical image segmentation models.

Feature	ProMISe	One-Prompt	FLanS	VoxelPrompt	SAT	Ours
	Li et al. [34]	Wu and Xu [35]	Da et al. [32]	Hoopes et al. [33]	Zhao et al. [31]	
Primary Prompt Type	Point (1 per vol.)	Click/BBox/Doodle/ SegLab	Free-form NL (anatomy-informed and agnostic)	NL → code	Medical terminology (text)	Free-form NL (clinician-style)
Modality Support	CT (colon/pancreas)	CT, MR, US, PET, Fundus, etc. (78 datasets)	CT (abdominal)	3D MRI/CT (neuro)	3D CT/MRI/PET (22 K scans, 72 datasets)	CT/MRI (2D axial)
Cross-Modal Fusion	Visual sampling + cross-attention	Prompt-Parser (Gaussian masking)	FiLM + CBN + SE/CBAM	Language-embedding conditioning + attention	Text-as-query (Transformer decoder)	FiLM + CBN + SE/CBAM (dynamic, multi-level)
Backbone	ViT-B (adapted SAM) + CNN	One-Prompt Former (novel)	MedSAM + CLIP text encoder	6-level 3D U-Net	3D U-Net encoder + Transformer decoder	Modified 2D U-Net + frozen BERT
Training Strategy	Partial finetune (adapters)	One-shot transfer (1 prompted sample)	3-stage (canonicalization → segmentation → alignment)	End-to-end from scratch (LLM + vision)	Contrastive knowledge pretrain + segmentation train	End-to-end (BERT frozen)
Loss Function Insight	Boundary-aware MSE + Dice/CE	Dice + CE	Dice + CE (+ classification for anatomy-informed)	Soft Dice + CE (code superv.)	Dice + CE	Task-dependent optimality: Dice (single-organ) vs. Jaccard (multi-organ)
Segmentation Scope	Binary tumor (colon/pancreas)	Arbitrary (1 organ or lesion)	24 abdominal organs (CT)	185+ neuroanatomy + 14 pathologies	497 classes (8 regions + lesions)	Brain tumors (3 classes) + CHAOS (4 organs)
Multi-Scan Support	Single volume	Single image	Single 2D slice	Arbitrary # of 3D volumes	Single 3D volume	Single 2D slice
Clinical Integration Focus	Tumor boundary precision	Annotation cost reduction	Zero-shot robustness to orientation	End-to-end workflow automation	Scalable grounding for LLMs	Clinician-in-the-loop, deployable real-time tool
Key Novelty	Boundary-aware loss + dual adapters	One-prompt transfer (1 sample → new task)	RAG + symmetry-aware canonicalization	Joint LLM + vision agent + native-res processing	Knowledge-enhanced text encoder + 497-class scale	Prompt-driven dynamic reconfiguration + task-dependent loss selection

**Table 10:** Quantitative comparison with foundation models on BraTS2021 (50 cases). Reported metrics: mean ± std. Dice (whole tumor), Hausdorff Distance (HD), and inference latency per 2D axial slice.

Method	Prompt Type	Dice (%)	HD (mm)	Time (ms/Slice)
MedSAM [24]	Tight bounding box	93.2±4.8	8.7±3.1	130
ProMISe [34]	1 point (3D volume)	94.5±3.9	6.9±2.7	95
FLanS [32]	“Segment glioma”	95.8±3.1	5.2±1.8	210
**Ours**	“Segment enhancing glioma”	97.7±1.2	3.2±1.5	42

## Data Availability

The data presented in this study are available in Kaggle at: https://www.kaggle.com/datasets/indk214/brain-tumor-dataset-segmentation-and-classification and in the Grand Challenge/Zenodo repositories at: https://chaos.grand-challenge.org/ and https://zenodo.org/records/3431873. These data were derived from the following publicly available resources: (1) Brain Tumor Dataset: Segmentation & Classification (Kaggle, accessed on 15 July 2025) and (2) CHAOS—Combined CT-MR Healthy Abdominal Organ Segmentation Challenge (Grand Challenge/Zenodo, accessed on 15 July 2025).
